# Supplementary material for: AgRP Neuron Activity Predicts and Tracks the Glycemic Response to Oral Glucose
Source: bioRxiv. 2026 Apr 3:2026.04.01.715678. Preprint. [Version 2] doi: 10.64898/2026.04.01.715678 (PMC13060230; doi:10.64898/2026.04.01.715678)
Supplement: Supplement 1 [file NIHPP2026.04.01.715678v2-supplement-1.pdf]

bioRxiv preprint doi: https://doi.org/10.64898/2026.04.01.715678; this version posted April 3, 2026. The copyright holder for this preprint (which was not certified by peer review) is the author/funder, who has granted bioRxiv a license to display the preprint in perpetuity. It is made available under aCC-BY 4.0 International license.

Supplemental figures

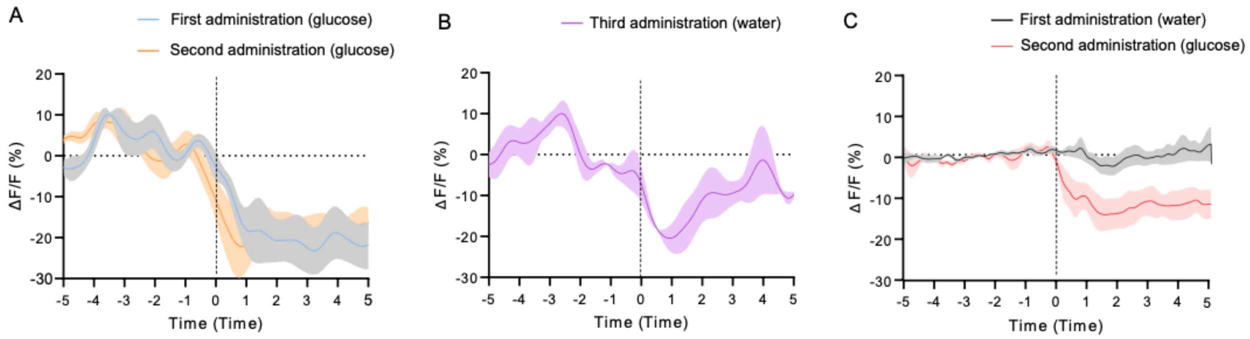

Supplementary Figure S1. AgRP neurons exhibit anticipatory responses to glucose administration via OG. (A) Mean AgRP neuron responses to the first and second OG glucose (2g/kg) (n = 3). (B) Mean AgRP neuron activity when water was administered after prior glucose exposures (n = 3). (C) AgRP neuron activity when water was given as the first OG, followed by 2.0 g/kg glucose on the subsequent gavage (n = 1).

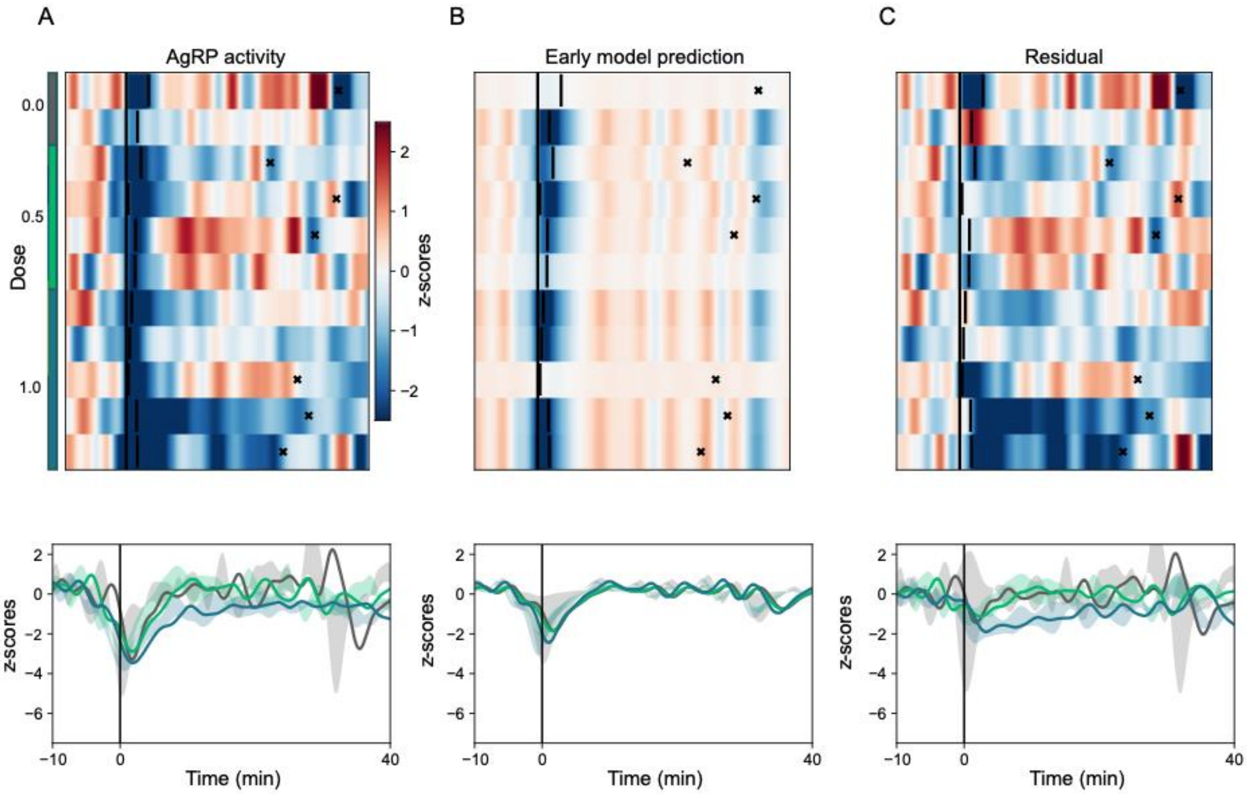

Supplementary Figure S2. Dissociation between phase 1 and phase 2 responses using an early-response model in low doses of glucose. (A-C), Heat-maps (top) and mean AgRP neuron activity (bottom) for the original (A), fitted (B), and residual (C) traces in mice administered with water (0.0), 0.5, or 1.0 g/kg glucose.

bioRxiv preprint doi: https://doi.org/10.64898/2026.04.01.715678; this version posted April 3, 2026. The copyright holder for this preprint (which was not certified by peer review) is the author/funder, who has granted bioRxiv a license to display the preprint in perpetuity. It is made available under aCC-BY 4.0 International license.

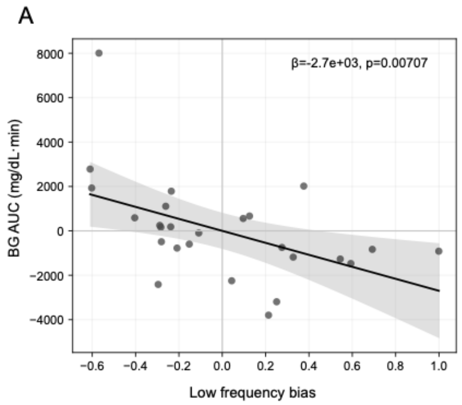

Supplementary Figure S3. Relationship between baseline low-frequency power and subsequent BG dynamics. (A), Relationship between individual session aperiodic slope in AgRP neuron activity (higher slope → greater low-frequency bias in baseline period) and incremental AUC of BG level. Slope from linear mixed effects model with corrections for OG dose and subject.

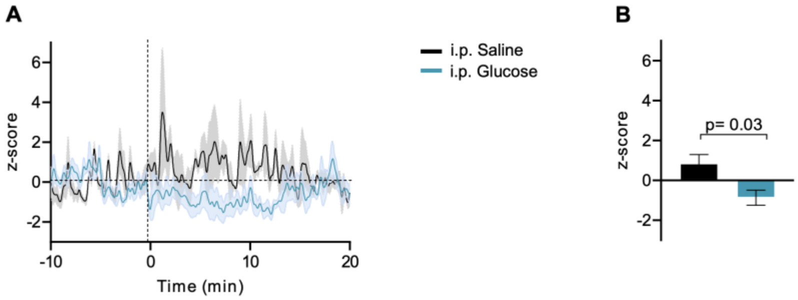

Supplementary Figure S4. AgRP neuron inhibition following i.p. injection of glucose (2g/kg) or saline. (A) GCaMP6s responses to i.p. glucose (2g/kg) or saline in 5-hour fasted mice. (B) Quantification of z-score from (A) over the 0-15 min period following injection. Mann-Whitney U, p=0.03, glucose: n = 5; saline: n=3.
